# Evolution of burnout and psychological distress in healthcare workers during the COVID-19 pandemic: a 1-year observational study

**DOI:** 10.1186/s12888-022-04457-2

**Published:** 2022-12-20

**Authors:** Samuel Cyr, Marie-Joelle Marcil, Cylia Houchi, Marie-France Marin, Camille Rosa, Jean-Claude Tardif, Stéphane Guay, Marie-Claude Guertin, Christine Genest, Jacques Forest, Patrick Lavoie, Mélanie Labrosse, Alain Vadeboncoeur, Shaun Selcer, Simon Ducharme, Judith Brouillette

**Affiliations:** 1 Research Centre, Montreal Heart Institute, 5000 Belanger street, Montreal, Québec, H1T 1C8 Canada; 2 Faculty of Pharmacy, Université de Montréal, P.O. Box 6128, Downtown Branch, Montreal, Québec, H3C 3J7 Canada; 3 Department of Psychiatry and Addiction, Université de Montréal, Roger-Gaudry Pavilion, Faculty of Medicine, P.O. Box 6128, Downtown Branch, Montréal, Québec, H3C 3J7 Canada; 4 Department of Psychology, UQAM, 100 Sherbrooke Street West, Montréal, Québec, H2X 3P2 Canada; 5 Research Centre, Institut universitaire en santé mentale de Montréal, 7331 Hochelaga Street, Montreal, Québec, H1N 3V2 Canada; 6 Montreal Health Innovations Coordinating Centre, 5000 Belanger street, Montreal, Québec, H1T 1C8 Canada; 7 Faculty of Medicine, Université de Montréal, P.O. Box 6128, Downtown Branch, Montreal, Québec, H3C 3J7 Canada; 8 Centre d’étude sur le Trauma, Research Centre, Institut universitaire en santé mentale de Montréal, 7331 Hochelaga Street, Montreal, Québec, H1N 3V2 Canada; 9 Faculty of Nursing, Université de Montréal, Marguerite-d’Youville Pavilion, P.O. Box 6128, Downtown Branch, Montreal, Québec, H3C 3J7 Canada; 10 Department of Organization and Human Resources, ESG UQAM, P.O. Box 8888, Downtown Branch, Montreal, Québec, H3C 3P8 Canada; 11 Department of Pediatrics, Division of Emergency Medicine, Centre Hospitalier Universitaire Sainte-Justine, 3175 Côte-Sainte-Catherine Road, Montreal, Québec, H3T 1C5 Canada; 12 Department of Psychiatry, Douglas Mental Health University Institute, McGill University, 6875 LaSalle Boulevard, Verdun, Québec, H4H 1R3 Canada; 13 McConnell Brain Imaging Centre, Montreal Neurological Institute, McGill University, 3801 University St, Montreal, Québec, H3A 2B4 Canada

**Keywords:** Burnout, Anxiety, Depression, Posttraumatic stress disorder, COVID-19, Health personnel

## Abstract

**Background:**

Long-term psychological impacts of the COVID-19 pandemic on healthcare workers remain unknown. We aimed to determine the one-year progression of burnout and mental health since pandemic onset, and verify if protective factors against psychological distress at the beginning of the COVID-19 pandemic (Cyr et al. in Front Psychiatry; 2021) remained associated when assessed several months later.

**Methods:**

We used validated questionnaires (Maslach Burnout Inventory, Hospital Anxiety and Depression and posttraumatic stress disorder [PTSD] Checklist for DSM-5 scales) to assess burnout and psychological distress in 410 healthcare workers from Quebec, Canada, at three and 12 months after pandemic onset. We then performed multivariable regression analyses to identify protective factors of burnout and mental health at 12 months. As the equivalent regression analyses at three months post-pandemic onset had already been conducted in the previous paper, we could compare the protective factors at both time points.

**Results:**

Prevalence of burnout and anxiety were similar at three and 12 months (52% vs. 51%, *p* = 0.66; 23% vs. 23%, *p* = 0.91), while PTSD (23% vs. 11%, *p* < 0.0001) and depression (11% vs. 6%, *p* = 0.001) decreased significantly over time. Higher resilience was associated with a lower probability of all outcomes at both time points. Perceived organizational support remained significantly associated with a reduced risk of burnout at 12 months. Social support emerged as a protective factor against burnout at 12 months and persisted over time for studied PTSD, anxiety, and depression.

**Conclusions:**

Healthcare workers’ occupational and mental health stabilized or improved between three and 12 months after the pandemic onset. The predominant protective factors against burnout remained resilience and perceived organizational support. For PTSD, anxiety and depression, resilience and social support were important factors over time.

**Supplementary Information:**

The online version contains supplementary material available at 10.1186/s12888-022-04457-2.

## Background

The COVID-19 pandemic is unprecedented in recent history, both in duration and number of cases and deaths. Having started nearly three years ago (in early 2020 [1]), the pandemic has become an ongoing reality for healthcare workers who have been on the frontline ever since. In our previously published study [2], as well as in systematic reviews/meta-analyses [3–16], it was found that the pandemic exposed healthcare workers to higher burnout (≈50%) as well as posttraumatic stress disorder (PTSD; ≈25%), anxiety (≈25%), and depression (≈15%). However, psychological distress among healthcare workers was already present before the pandemic started [17–19]. In our multivariable model [2] using data measured three months after the pandemic onset, resilience and perceived organizational support were two protective factors significantly associated with lower odds of burnout and lower scores of psychopathological symptoms. Other related factors have also been documented at the beginning of the pandemic, such as higher exposure to the COVID virus, social support, and personal protective equipment (PPE) availability [20–22].

The pandemic can no longer be recognized as an acute stressor but rather as a chronic stressor, with associated issues such as a higher prevalence of psychopathology potentially emerging [23]. More than 1000 studies have been conducted on the psychological health of healthcare workers since the beginning of this pandemic. However, most were conducted in the first six months of the pandemic. As it is still ongoing, there is a need to continue monitoring the long-term evolution of this population’s mental health. This is particularly relevant considering that previous pandemics (including the 2003 SARS outbreak), although less important in terms of cases/deaths and duration than the COVID-19 pandemic, had a long-term impact on the psychological health of healthcare workers [24]. Higher perceived stress still present one year after the onset of the SARS pandemic in high-risk health care workers was a particular example of a long-term effect on psychological health [25]. To our knowledge, few research teams have attempted to verify whether the factors associated with burnout and psychopathological symptoms found at the beginning of the COVID-19 pandemic remained associated with the same outcomes when assessed several months later.

The objectives of this study were to measure the evolution of burnout and PTSD, anxiety and depressive symptoms in healthcare workers at 3 and 12 months after the COVID-19 pandemic onset, and to determine if individual, occupational, social, and organizational factors previously associated, or not, three months after the onset of the pandemic [2] remained associated, or not, with the outcomes 12 months after it began.

## Methods

### Setting, patients and study design

We conducted this observational study at the Montreal Heart Institute (Quebec, Canada). The project was approved by both the scientific and ethics committees on May 14th, 2020. All methods were performed in accordance with the relevant guidelines and regulations and as per the approved protocol. Informed consent was obtained from all subjects of this study. This study’s complete and detailed methodology has already been published [2].

Through Quebec associations of health professionals, traditional media and social media accounts managed by collaborators of the study, we circulated a newsletter explaining the objectives of the study and referring to a web page where we verified the eligibility of interested healthcare workers. We recruited 564 healthcare workers across Quebec between May 21st and June 5th, 2020. Our sample completed two electronic surveys. First, they completed a survey three months after the start of the COVID-19 pandemic in Quebec (March 2020), in June 2020, with a response rate of 83%: the results of this survey are already published [2]. Then, we assessed the outcomes and exposures again at 12 months post-start of the pandemic, in March 2021.

We included healthcare workers in clinical and non-clinical settings, such as administrative agents, beneficiary attendants, physicians/residents, laboratory technicians, kitchen attendants, maintenance agents, managers, nurses, or other healthcare professionals (e.g., occupational therapists, respiratory therapists, nutritionists, psychologists, social workers).

At the end of February 2021, we sent up to three e-mails to invite participants to complete the 12-month online survey. Participants had one week (from March 15th to March 22nd) to answer the survey. We accepted partially completed questionnaires. However, depending on the questionnaire, a maximum of 3 missing values were accepted and imputed using the average of the non-missing values [2]. For the regression analyses, no imputation was done for missing variables.

### Measurements/outcomes

All measurements and outcomes were measured at both time points (3-month and 12-month). We assessed burnout as a categorical variable (present or not present) with the Maslach Burnout Inventory (MBI-2). The MBI-2 measures two dimensions of burnout syndrome, namely emotional exhaustion, with a feeling of being burned out, and depersonalization, with a sensation of indifference to the feelings or suffering of other people. Experiencing at least weekly emotional exhaustion and/or depersonalization was considered clinically relevant burnout symptoms [26, 27]. The three psychopathologies studied, namely PTSD, anxiety, and depressive symptoms, were assessed as continuous scores with respectively, the PTSD Checklist for DSM-5 (PCL-5, a 20-item questionnaire rated on a 5-point Likert scale, with scores ranging from 0 to 80), the Hospital Anxiety and Depression Scale (HADS-A and HADS-D, each subscales comprising 7 items, with subscale total scores going from 0 to 21). The time frame of the mental health measures was in the past month for the PCL-5 and in the past week for both subscales of the HADS. The presence of symptoms of PTSD, anxiety, and depression was defined as a score of 31 or more for PCL-5 [28–30] and 11 or more for each HADS subscales [31].

For the other factors studied, we have previously published a summary of all measurements [2]. In brief, resilience was assessed using the self-rated 10-item Connor-Davidson Resilience Scale (CD-RISC-10), a 10-item scale rated on a 5-point Likert scale (0—not true at all to 4—true nearly all of the time), with total scores ranging from 0 to 40 [32]. The 6-item Social Support Questionnaire (SSQ6) was used to measure satisfaction with social support. The 6 items are rated on a 6-point Likert scale (1—very dissatisfied to 6—very satisfied), with the total scores varying from 6 to 36 [33]. Finally, we used the 8-item Perceived Organizational Support (POS) Scale to assess our respondents’ perceived organizational support. The 8 items are rated on a 7-point Likert scale (1—strongly disagree to 7—strongly agree), with a total score on this scale that can range up from 0 to 48 [34]. For all these questionnaires, a higher score indicated a greater importance of the factor and each score was assessed as a continuous variable in the analysis. The survey additionally verified workload, access to simulation-based education, access to mental health help, and access to personal protective equipment (PPE) and feeling of security using PPE.

The detailed calculation of sample size may be found in our previous publication [2]. Briefly, a sample size of 285 participants was determined to provide a power of 80% to detect an odds ratio of 0.72 for a one-standard-deviation increase in resilience, using a two-sided 0.05 significance level and assuming a rate of burnout of 50%.

### Statistical analyses

Participant characteristics were summarized using counts and percentages for categorical variables and mean ± standard deviation (*SD*) for continuous variables. Comparisons between 3 months and 12 months for outcomes (burnout, PTSD, anxiety and depression) and risk/protective factors were made using McNemar tests, paired Student t-tests or Wilcoxon signed-rank tests. Multivariable logistic and linear regression were done to assess the association between risk/protective factors and outcomes measured at 12 months, using the same approach as the one described in our previously published paper [2]. Briefly, the models included pre-specified independent variables and were adjusted for pre-specified adjustment variables using a stepwise procedure. The adjusted odds ratio for logistic regressions and adjusted coefficients for linear regressions were calculated with 95% confidence intervals. An exploratory analysis was conducted for each outcome by adding self-compassion into the final multivariable model. No imputation was done for missing data. A *p*-value < 0.05 was considered statistically significant. All statistical analyses were performed with SAS release 9.4 [SAS Institute Inc., Cary, NC, USA].

## Results

Of the 564 initially recruited participants, 467 completed the 3-month [2] and 410 the 12-month survey, resulting in 83 and 73% response rates, respectively. Of those, 394 participants had responded to both surveys and will be referred to as “3- & 12-month surveys responders”. Socio-demographic, occupational data, and COVID-19 specific characteristics of the 12-month survey responders are presented in Table 1. These characteristics were similar to those of the “3- & 12-month surveys responders” (Table S1 in Supplementary Material) and those of the original cohort (*n* = 467) [2]. Participants were 40 years old on average (*SD* = 9), mainly of the female sex (91%), and Caucasian (95%). One year after the pandemic, most participants (95%) still worked in the healthcare system. Participants worked as other health professionals (e.g., nutritionists, occupational and respiratory therapists; 32%), physicians (25%) or nurses (22%), and 93% felt they had access to mental help if needed.

**Table 1:** Socio-demographic, occupational data, COVID-19 specific characteristics of participants 12 months after the COVID-19 pandemic onset *(12-month survey responders, n = 410)*

Variables	Mean ± *SD* or *n* (%)	All *n* = 410
Age (years)	40 ± 9	409
Sex (female)	366 (90.6%)	404
Ethnicity		410
Caucasian	389 (94.9%)	
Hispanic	2 (0.5%)	
Black	4 (1.0%)	
Asian	7 (1.7%)	
Native American	1 (0.2%)	
Two of the above	7 (1.7%)	
Marital status		408
Never married	69 (16.9%)	
Married/Re-married	120 (29.4%)	
Separated/Divorced	26 (6.4%)	
Common-law union	184 (45.1%)	
Widowed	1 (0.2%)	
Other	8 (2.0%)	
Parental status (yes)	262 (64.1%)	409
Antecedent of psychiatric disorder (yes)	119 (29.0%)	410
Work type		409
Administrator	18 (4.4%)	
Administrative agent	15 (3.7%)	
Beneficiary attendant	8 (2.0%)	
Laboratory technician/technologist	8 (2.0%)	
Nurse	89 (21.8%)	
Other health professional (ergotherapist, respiratory therapist, psychologist, social worker, etc.)	129 (31.5%)	
Paramedics	9 (2.2%)	
Physician	102 (24.9%)	
Resident physician	7 (1.7%)	
Other	24 (5.9%)	
Workplace		407
Community clinic	51 (12.5%)	
Nursing home	21 (5.2%)	
University hospital	126 (31.0%)	
Non-University hospital	73 (17.9%)	
Medical clinic	33 (8.1%)	
Other	103 (25.3%)	
Intensive care or emergency work	58 (14.3%)	407
Workload (hours/week)		375
≤ 34	79 (21.1%)	
35–44	207 (55.2%)	
45–54	60 (16.0%)	
55–64	14 (3.7%)	
≥ 65	15 (4.0%)	
Current work status		394
Still employed in the Quebec health system	375 (95.2%)	
Employee of another employer	5 (1.3%)	
Self-employed	2 (0.5%)	
Unemployed	1 (0.3%)	
Student	2 (0.5%)	
Retired	2 (0.5%)	
Other	7 (1.8%)	
Access to mental help (yes)	380 (93.4%)	407
Type of mental help professional		380
Psychologist	93 (24.5%)	
Psychotherapist	16 (4.2%)	
Social worker	7 (1.8%)	
Family doctor	49 (12.9%)	
Employee assistance program	182 (47.9%)	
Other	33 (8.7%)	
Access to PPE		404
Never or rarely	10 (2.4%)	
Sometimes	14 (3.5%)	
Often	75 (18.6%)	
Always	305 (75.5%)	
Perception of security using PPE		400
Totally safe	73 (18.3%)	
Pretty safe	293 (73.3%)	
Rather or totally in danger	34 (8.5%)	
Participation in simulation-based education (yes)	109 (26.9%)	405
Last simulation session		109
< 1 week	2 (1.8%)	
< 1 month	5 (4.6%)	
1–2 months ago	9 (8.3%)	
< 6 months	31 (28.4%)	
< 1 year	62 (56.9%)	
COVID status		408
Positive	1 (0.2%)	
Negative	346 (84.8%)	
Recovered	27 (6.6%)	
Never been tested	34 (8.3%)	
Direct COVID patient care (yes)	168 (41.3%)	407
Reassignment (yes)	139 (34.0%)	409

Data are presented as Mean ± *SD* or *n* (%)

*Abbreviations*: *PPE* personal protective equipment

Mean scores of resilience, satisfaction of social support, perceived organizational support, and self-compassion at three months and 12 months are presented in Table 2. Resilience (27.49 ± 6.16 vs. 27.45 ± 6.16, *p* = 0.91) and perceived organizational support scores (22.76 ± 11.42 vs. 23.47 ± 11.77, *p* = 0.12) remained stable over time. There was a non-significant upward trend in the level of satisfaction for social support between three and 12 months (28.79 ± 6.20 vs. 29.17 ± 6.01, *p* = 0.07), while there was a small but significant increase in self-compassion scores over time (12.10 ± 3.64 vs. 12.73 ± 3.61, *p* < 0.0001).

**Table 2:** Psychological questionnaire scores of participants 3 and 12 months after the COVID-19 pandemic onset *(3- & 12-month surveys responders, n = 394)*

Psychological scores	3 months		12 months		*p*
	Mean ± *SD*	*N*	Mean ± *SD*	*N*	
Resilience (CD-RISC-10; 0–40)	27.49 ± 6.16	392	27.45 ± 6.16	390	0.91
Satisfaction of social support (SSQ6-S; 6–36)	28.79 ± 6.20	389	29.17 ± 6.01	380	0.07
Perceived organizational support (SPOS8; 0–48)	22.76 ± 11.42	391	23.47 ± 11.77	379	0.12
Self-compassion (SCS; 3–21)	12.10 ± 3.64	391	12.73 ± 3.61	378	<.0001

*P*-values were generated from a paired t-test for resilience, perceived organizational support, and self-compassion, and from Wilcoxon signed-rank test for social support satisfaction scores

*Abbreviations*: *CD-RISC-10* 10-item Connor-Davidson Resilience Scale, *SCS* Self-compassion scale, *SD* Standard derivation, *SPOS8* 8-item Survey of Perceived Organizational Support, *SSQ6-S* Social Support Questionnaire Short form - Satisfaction with social support perceived

**Fig. 1:**
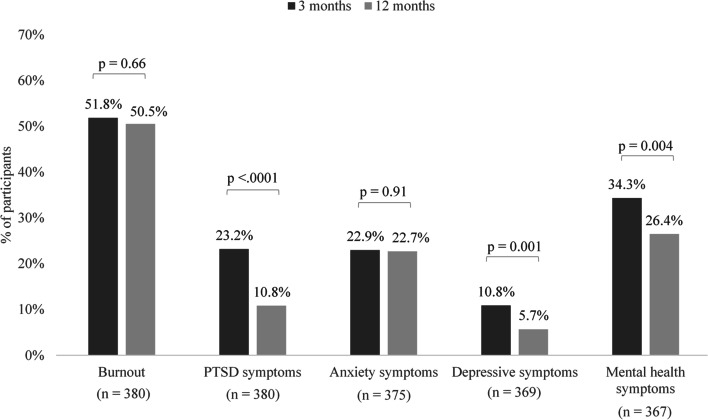
Evolution of mental distress at 3 and 12 months after the onset of COVID-19 pandemic (*3- & 12-month surveys responders n = 394). Notes:* Presence of burnout is defined as feeling emotional exhaustion or depersonalization at least weekly on MBI-2. Presence of PTSD, anxiety, and depressive symptoms are defined as PCL-5 ≥ 31, HADS-A ≥ 11, and HADS-D ≥ 11, respectively. Any mental health symptoms are defined as having one or more symptoms of PTSD, anxiety, or depression. Abbreviations: MBI-2, Maslach Burnout Inventory; PTSD, Posttraumatic stress disorder; HADS, Hospital Anxiety and Depression Scale; PCL-5, Posttraumatic Stress Disorder Checklist for DSM-5

Evolution in the prevalence of burnout and symptoms of any psychopathology measured in the 3- & 12-month surveys responders are presented in Fig. 1. There was no significant difference in proportions of burnout (52% vs. 51%, *p* = 0.66) and anxiety (23% vs. 23%, *p* = 0.91) between 3 months and 12 months after the pandemic onset. From three months to 12 months, 131 participants (34%) remained burned out, 61 (16%) had a new onset of burnout, burnout resolved in 66 (17%), and 122 (32%) continued to be burnout-free. The proportion of participants with symptoms of PTSD (23% vs. 11%, *p* < 0.0001), of depression (11% vs. 6%, *p* = 0.001), or any mental health symptoms (34% vs. 26%, *p* = 0.004) decreased significantly over time.

Table 3 presents the 12-month multivariable logistic regression model for burnout. Higher resilience (OR = 0.66, 95% CI [0.50, 0.87], *p* = 0.003), social support (OR = 0.71, 95% CI [0.54, 0.93], *p* = 0.01), and perceived organizational support (OR = 0.66, 95% CI [0.51, 0.85], *p* = 0.001) were significantly associated with lower odds of burnout at 12 months. Tables 4, 5 and 6 show the 12-month multivariable linear regression models for PTSD, anxiety, and depressive symptoms. Higher resilience and social support were significantly associated with lower PTSD scores (Est = − 0.29, 95% CI [− 0.45, − 0.12], *p* = 0.0007; Est = −0.26, 95% CI [−0.42, −0.10], *p* = 0.001), anxiety (Est = −1.35, 95% CI [−1.77, −0.94], *p* < 0.0001; Est = −0.68, 95% CI [−1.08, −0.28], *p* = 0.0009), and depressive symptoms (Est = −1.11, 95% CI [−1.43, −0.78], *p* < 0.0001; Est = −0.91, 95% CI [−1.23, −0.59], *p* < 0.0001). The security perception of being “rather or totally at danger” using PPE was significantly associated with increased PTSD symptoms (Est = 1.21, 95% CI [0.57, 1.85], *p* = 0.0002) when compared with the perception of being “totally safe”. A prior history of psychiatric disorder was significantly associated with more PTSD (Est = 0.47, 95% CI [0.14, 0.79], *p* = 0.005) and anxiety symptoms (Est = 1.16, 95% CI [0.34, 1.97], *p* = 0.006).

**Table 3:** Adjusted odds ratios, 95% confidence interval and *p*-values from multivariable logistic regression model for burnout symptoms measured at 12-month among healthcare workers *(12-month survey responders, n = 336; 74 missings)*

Variables		OR	95% CI		*p*
Independent	Resilience	0.66	0.50	0.87	0.003
	Social support	0.71	0.54	0.93	0.01
	Workload				0.13
	[35–44] h vs ≤ 34 h	1.65	0.89	3.06	0.11
	[45–54] h vs ≤ 34 h	1.82	0.81	4.10	0.15
	[55–64] h vs ≤ 34 h	3.67	0.86	15.66	0.08
	≥ 65 h vs ≤ 34 h	4.82	1.11	20.95	0.04
	Perceived organizational support	0.66	0.51	0.85	0.001
	Access to simulation based education (yes vs no)	0.89	0.52	1.51	0.66
	Access to mental health help (yes vs no)	0.74	0.25	2.18	0.58
	Access to PPE				0.39
	Sometimes vs Never or rarely	7.01	0.56	88.16	0.13
	Often vs Never or rarely	4.69	0.69	31.91	0.11
	Always vs Never or rarely	3.79	0.58	24.78	0.16
	PPE perception of security				0.29
	Pretty safe vs Totally safe	1.60	0.85	3.02	0.15
	Rather or totally in danger vs Totally safe	1.98	0.66	5.93	0.23
Adjustment	Psychiatric antecedent (yes vs no)	1.82	1.06	3.15	0.03

ORs are presented for an increase of one standard deviation (*SD*) for continuous variables (resilience; *SD* = 5.73, social support; *SD* = 5.69, and perceived organizational support; *SD* = 11.53). Type of employment and work environment were considered in the stepwise but not selected. The final model is therefore the one above

*Abbreviations*: *CI* Confidence intervals, *OR* Odds ratio, *PPE* Personal protective equipment

**Table 4:** Adjusted coefficient, 95% confidence interval and *p*-values from multivariable linear regression model for posttraumatic stress symptoms measured at 12-month among healthcare workers *(12-month survey responders, n = 343; 67 missings)*

Variables		Coefficient	95% CI		*p*
Independent	Resilience	−0.29	−0.45	−0.12	0.0007
	Social support	−0.26	−0.42	−0.10	0.001
	Workload				0.50
	[35–44] h vs ≤ 34 h	−0.25	−0.62	0.12	0.19
	[45–54] h vs ≤ 34 h	0.03	−0.45	0.52	0.89
	[55–64] h vs ≤ 34 h	0.08	−0.74	0.89	0.85
	≥ 65 h vs ≤ 34 h	0.02	−0.78	0.83	0.95
	Perceived organizational support	−0.06	−0.21	0.10	0.48
	Access to simulation based education (yes vs no)	−0.03	−0.36	0.29	0.83
	Access to mental health help (yes vs no)	0.30	−0.34	0.95	0.35
	Access to PPE				0.21
	Sometimes vs Never or rarely	0.37	−1.01	1.75	0.60
	Often vs Never or rarely	−0.43	−1.49	0.62	0.42
	Always vs Never or rarely	−0.57	−1.59	0.46	0.28
	PPE perception of security				0.0008
	Pretty safe vs Totally safe	0.25	−0.14	0.63	0.21
	Rather or totally in danger vs Totally safe	1.21	0.57	1.85	0.0002
Adjustement	Psychiatric antecedent (yes vs no)	0.47	0.14	0.79	0.005

Regression coefficients are presented for an increase of one standard deviation (*SD*) for continuous variables (resilience; *SD* = 6.09, social support; *SD* = 5.93, perceived organizational support; *SD* = 11.65). 12-month responders had a mean score of 15.28 ± 12.04 on the PCL-5 scale, *n* = 397. Type of employment and sex were considered in the stepwise but not selected. The final model is therefore the one above

*Abbreviations*: *CI* Confidence intervals, *PPE* Personal protective equipment, *PCL-5* Posttraumatic Stress Disorder Checklist for DSM-5

**Table 5:** Adjusted coefficient, 95% confidence interval and *p*-values from multivariable linear regression model for anxiety symptoms measured at 12-month among healthcare workers *(12-month survey responders, n = 341; 69 missings)*

Variables		Coefficient	95% CI		*p*
Independent	Resilience	−1.35	−1.77	−0.94	<.0001
	Social support	−0.68	−1.08	−0.28	0.0009
	Workload				0.26
	[35–44] h vs ≤ 34 h	−0.03	−0.97	0.91	0.95
	[45–54] h vs ≤ 34 h	0.67	−0.55	1.89	0.28
	[55–64] h vs ≤ 34 h	0.58	−1.46	2.62	0.58
	≥ 65 h vs ≤ 34 h	1.91	−0.18	4.00	0.07
	Perceived organizational support	−0.09	−0.49	0.30	0.64
	Access to simulation based education (yes vs no)	0.33	−0.51	1.18	0.44
	Access to mental health help (yes vs no)	0.34	−1.28	1.97	0.68
	Access to PPE				0.52
	Sometimes vs Never or rarely	2.07	−1.41	5.54	0.24
	Often vs Never or rarely	0.75	−1.91	3.40	0.58
	Always vs Never or rarely	0.39	−2.19	2.97	0.77
	PPE perception of security				0.054
	Pretty safe vs Totally safe	0.36	−0.60	1.32	0.46
	Rather or totally in danger vs Totally safe	1.93	0.33	3.53	0.02
Adjustment	Intensive care of emergency work (yes vs no)	1.28	0.23	2.33	0.02
	Psychiatric antecedent (yes vs no)	1.16	0.34	1.97	0.006

Regression coefficients are presented for an increase of one standard deviation (*SD*) for continuous variables (resilience; *SD* = 6.09, social support; *SD* = 5.93, perceived organizational support; *SD* = 11.65). 12-month responders had a mean score of 7.32 ± 3.88 on the anxiety subscale of the HADS, *n* = 398

*Abbreviations*: *CI* Confidence intervals, *HADS* Hospital Anxiety and Depression Scale, *PPE* Personal protective equipment

**Table 6:** Adjusted coefficient, 95% confidence interval and *p*-values from multivariable linear regression model for depression symptoms measured at 12-month among healthcare workers *(12-month survey responders, n = 341; 69 missings)*

Variables		Coefficient	95% CI		*p*
Independent	Resilience	−1.11	−1.43	−0.78	<.0001
	Social support	−0.91	−1.23	−0.59	<.0001
	Workload				0.06
	[35–44] h vs ≤ 34 h	−0.26	−1.03	0.50	0.50
	[45–54] h vs ≤ 34 h	0.66	−0.33	1.65	0.19
	[55–64] h vs ≤ 34 h	0.03	−1.63	1.69	0.97
	≥ 65 h vs ≤ 34 h	1.62	−0.01	3.26	0.052
	Perceived organizational support	−0.29	−0.61	0.02	0.07
	Access to simulation based education (yes vs no)	−0.23	−0.90	0.43	0.49
	Access to mental health help (yes vs no)	0.60	−0.71	1.91	0.37
	Access to PPE				0.11
	Sometimes vs Never or rarely	1.86	−0.96	4.68	0.20
	Often vs Never or rarely	−0.44	−2.58	1.71	0.69
	Always vs Never or rarely	−0.63	−2.73	1.46	0.55
	PPE perception of security				0.33
	Pretty safe vs Totally safe	0.38	−0.40	1.16	0.34
	Rather or totally in danger vs Totally safe	0.97	−0.32	2.26	0.14

Regression coefficients are presented for an increase of one standard deviation (*SD*) for continuous variables (resilience: *SD* = 6.09, social support: *SD* = 5.93, perceived organizational support: *SD* = 11.65). 12-month responders had a mean score of 4.69 ± 3.45 on the depression subscale of the HADS, *n* = 396. Psychiatric history was considered in the stepwise but not selected. The final model is therefore the one above

*Abbreviations*: *CI* Confidence intervals, *HADS* Hospital Anxiety and Depression Scale, *PPE* Personal protective equipment

We examined if the factors found in the 12-month multivariable regression models were the same as those previously identified at three months [2]. Table 7 compares factors significantly associated with burnout and symptoms of PTSD, anxiety, and depression 3 and 12 months after the pandemic onset. Higher resilience was associated with lower odds of burnout and lower symptoms of PTSD, anxiety, and depression at both time points. Social support generally remained associated with psychopathologies across time and emerged as a new protective factor against burnout at the 12-month time point. Higher perceived organizational support was associated with lower odds of burnout and lower symptoms of other psychopathologies at three months, but was only linked with burnout at 12 months. PPE perception of security remained associated with PTSD symptoms at both time points.

**Table 7:** Summary table comparing factors significantly associated with burnout status, posttraumatic stress disorder, anxiety and depression symptoms among healthcare workers at 3 and 12 months after the COVID-19 pandemic onset *(3-month survey responders column, extracted from the original article published on Front Psychiatry* [2] *and 12-month survey responders)*

Dependent variables									
		Burnout		PTSD		Anxiety		Depression	
		3	12	3	12	3	12	3	12
Independent variables	Resilience	Y	Y	Y	Y	Y	Y	Y	Y
	Social support	N	Y	Y	Y	Y	Y	Y	Y
	Workload	N	N	N	N	N	N	N	N
	Perceived organizational support	Y	Y	Y	N	Y	N	Y	N
	Access to simulation based education	N	N	N	N	N	N	N	N
	Access to mental health help	N	N	N	N	N	N	N	N
	Access to PPE	N	N	N	N	N	N	N	N
	PPE perception of security	N	N	Y	Y	N	N	N	N
Adjustment variables	Type of employment	–	N	Y	N	–	–	Y	–
	Intensive care or emergency work	–	–	–	–	–	Y	–	–
	Direct COVID care	–	–	–	–	–	–	–	–
	Reassignment	–	–	–	–	–	–	Y	–
	Participant’s COVID status	–	–	–	–	–	–	–	–
	Sex	–	–	–	N	–	–	–	–
	Work environment	–	N	–	–	Y	–	–	–
	Psychiatric antecedents	–	Y	Y	Y	Y	Y	Y	N

Abbreviations: *N* Not significant, *PPE* Personal protective equipment, *PTSD* posttraumatic stress disorder, *Y* Yes (statistically significant *p* < 0.05), – adjustment variable not selected in the stepwise procedure (*p* ≥ 0.02)

We added the variable self-compassion to our 12-month models in an exploratory analysis. On top of the other factors included in the model, self-compassion was significantly negatively associated with PTSD (Est = −0.21, 95% CI [−0.38, −0.04], *p* = 0.02) and depressive symptoms (Est = −0.36, 95% CI [−0.71, −0.01], *p* = 0.04) one year after the pandemic onset. Self-compassion was not associated with burnout or anxiety measured at 12 months. Complete information for this exploratory analysis may be found in Tables S2 to S5 of supplementary material.

## Discussion

This study is among the first to assess the one-year evolution of psychological health and its contributing or protective factors in healthcare workers facing the COVID-19 pandemic. A year after the pandemic onset, healthcare workers’ mental health did not deteriorate further. Indeed, burnout and anxiety were stable, while PTSD and depression significantly decreased. Even if the global rate of burnout was stable, it did not reflect solely a chronic illness in specific participants. Indeed, an equal number had their burnout resolved, and another had new onset. Resilience and perceived organizational support were persistent protective factors against burnout at 12 months, while social support and resilience were constant factors over time for psychopathological outcomes. Compared to the 3-month time point, self-compassion emerged as a protective factor against PTSD and depression at 12 months.

Because the epidemiological situation related to the COVID-19 pandemic fluctuates significantly, it is relevant to compare the epidemiology at the time of the two surveys. In June 2020, when the first survey was administered, the peak of the first wave of COVID-19 in Quebec had just been passed, with 7630 active cases during the month. At the time of the second survey, in March 2021, Quebec had again just passed the peak of a wave, the second wave this time, with a total of 10,343 active cases during the month. In terms of hospitalizations, the data are similar between the two surveys, with slightly more hospitalizations at the time of the 12-month survey than at that of the 3-month survey, with 579 hospitalizations in June 2020 and 1178 in March 2021 [35].

While very encouraging, the global stability and, in some disorders, a reduction of mental health adverse outcomes for healthcare workers is relatively surprising, given that the pandemic is still ongoing. Various hypotheses may help understand this finding. First, healthcare workers may have gradually grown accustomed to the pandemic conditions due to its prolonged nature. This concept is known as habituation and has been described in the general population and healthcare workers in the COVID-19 context [36–39]. Secondly, systemic desensitization may help explain the decrease in PTSD symptoms. In this case, repetitive exposures to the stressor COVID-19 may eventually reduce fear or lead to deconditioning. Repetitive exposure will gradually associate the stressful event with better working conditions and a more confident and reassuring context to move away from the pathological anxious reaction present in PTSD [40, 41]. Finally, the development of the COVID-19 vaccine offered substantial hope for the end of this health crisis [42], and hope is a contributing factor in the recovery from mental health illness [43, 44]. In addition, the vaccine represents protection from the virus, helping healthcare workers feel safer while performing their duties. This may explain the reduction of PTSD symptoms at the 12-month survey, as workers may have felt less threatened in their physical integrity. We note that the Quebec vaccination campaign was already underway at the 12-month survey, and approximately 50,000 healthcare workers had received a first dose at that point [45]. Since resilience and perceived organizational support remained stable over time, these are probably not the driver of the reduction in PTSD or depressive symptoms. Self-compassion did increase over time and was significantly associated with lower odds of PTSD and depressive symptoms, the two psychopathologies with improvement over time. This is in line with a meta-analysis that identified a large negative effect size of an increase in self-compassion on the decrease in psychopathologies [46]. Also, it is interesting to note the stability in the symptoms of burnout compared to the reduction in symptoms of certain mental disorders, including depression. This could be explained by the purely professional roots of burnout, as opposed to the more global bio-psycho-social roots of depression, and COVID-19 being an ongoing active workplace-related stressor [47, 48]. This different evolution also reinforces the idea that burnout and depression remain two distinct entities, even though they may overlap.

To our knowledge, only a few studies have verified more than once the psychological symptoms of healthcare workers during this pandemic, and those were primarily performed in the first weeks or months of the pandemic [49–62]. As a result, the currently available literature does not allow us to make proper comparisons. Nevertheless, Ercolani et al. [61], compared the prevalence of burnout at the beginning of the pandemic and one year later, in a similar way to our study. Although the population differed from ours, focusing on healthcare workers in palliative care, the symptoms of burnout, while much lower than the levels found in our population, were similar between the two time points (22.0% vs 24.1%, *p* = 0.666). On the contrary, Lasalvia et al. [63] also verified the evolution of mental health symptoms one year after the pandemic, but their results diverged from ours. Indeed, they determined that all the mental health disorders studied (burnout, anxiety, and depression) had increased between the beginning of the pandemic and 12 months later, except PTSD, which was stable. None of these outcomes have increased in our study, and some were stable or decreased over time. Our results seem to agree better with those of Chew et al., who found reduced stress levels three months after the pandemic compared to the beginning of the pandemic [49]. Although we have not verified healthcare workers’ perceived stress, our results showed stability in anxiety symptoms and a significant decrease in symptoms of PTSD. Teo et al. [56] observed an increase in burnout each month following the pandemic from March 2020 (3-month) to August 2020 (8-month), which contrasts with the stability we observed. Those investigators determined that anxiety remained unchanged over time, which is more consistent with our study. In another contrast with our findings, Lopez Steinmetz et al. [51] observed a greater proportion of healthcare workers with symptoms of depression, anxiety, or any mental disorder, from the first to the fourth month into the pandemic. We note that the different observations across studies may be attributed to the various locations and COVID-19-related circumstances (distinct COVID-19 incidence, pressures on the health care system, economic factors, etc.) in these locations.

The second objective of our study was to verify if the associations of factors with psychological distress three months after COVID-19 onset persisted a year after the start of the pandemic [2]. We found that perceived organizational support remained an important protective factor against burnout but lost significance for the three psychopathologies. This could indicate that perceived organizational support significantly impacted PTSD, anxiety, and depression at the beginning of the pandemic (3 months) [2, 64–66], especially with the important perturbation that occurred at the organizational level but had a lesser impact as the pandemic progressed. Social support offers durable protection in time for these three psychopathologies, as it appeared at three and 12 months. Social support did not appear as a protective factor against burnout at three months, in contrast to its significant effect at 12 months. We hypothesize that in the early months of the pandemic (at the 3-month survey), perceived organizational support and/or resilience have a heightened importance for burnout. Once the pandemic persists for a longer period of time (12-month survey), social support becomes factor of importance in burnout alongside perception of organizational support and resilience. Resilience was a stable protective factor over time for all outcomes. PPE perception of security manifested across time for PTSD. Considering that PTSD could arise from the fear for one’s safety [67], being confident about the protection used against the virus is relevant. Finally, a year after the pandemic onset, psychiatric antecedents continued to be associated with PTSD and anxiety at 12 months. Given that the pandemic is still ongoing, it will be necessary to continue monitoring healthcare workers’ psychological symptoms. Following the end of the health crisis, the persistence of symptoms should be kept under surveillance. The results of this study allow us to hypothesize that burnout may still be a burden while psychological suffering could alleviate with time. Also, this study identified several protective factors that persisted between three and 12 months after the beginning of the pandemic. It would be relevant to verify whether an intervention that aims to increase resilience or perceived organizational support would impact burnout while those focusing on social support and self-compassion would reduce PTSD or depressive symptoms.

Our study has limitations, as previously published [2]. We could not establish a temporal link between the factors and outcomes, given that they were measured at the same time [68]. The web-based aspect of the study might have caused a volunteer and selection bias [69]. Additionally, because this study presented the evolution of burnout and psychopathologies over time, and that we obtained a lower response rate for the 12-month survey than the one at three months, this resulted in a smaller sample size. Although this sample size provided sufficient power for our primary objective, it may not have been adequate for secondary or exploratory objectives. Nevertheless, the results for several secondary endpoints were significant. Also, we are aware of the inherent limitation of the PCL-5 scale, which assesses recognized symptoms of PTSD but does not determine the presence of a qualifying traumatic event. Despite continuing debate, as presented in the work of North et al. [70], some current literature since the onset of the COVID-19 pandemic recognizes the pandemic as a traumatic event for healthcare workers [71–73]. Because of this appreciation of the traumatic aspect of the pandemic, the terminology of PTSD symptomatology related to the use of the PCL-5 scale was used in this work. Finally, we note that our population is predominantly female between 30 and 50 years of age. However, despite this overrepresentation of women in our study and middle-aged participants, it should be noted that the population is, in fact, representative of the local healthcare worker population, which has been identified as 82% female with a young age average, according to a recent Quebec government survey [74].

## Conclusions

In conclusion, this study presents encouraging results for healthcare workers facing COVID-19, with mental health symptoms either stabilizing (for burnout and anxiety) or decreasing (for PTSD, depression, and any mental disorders) between three and 12 months after the onset of the pandemic. We also documented how different protective factors are associated with these mental health outcomes over time. Resilience and perceived organizational support remained the predominant protective factors at 12 months against burnout, whereas social support and resilience were persistent factors over time against psychopathological outcomes. Self-compassion emerged as an influential protective factor against PTSD and depression. Though our data is based on a convenience sample and may not be generalizable beyond Quebec, these findings could guide the development of other research projects regarding the psychological and occupational health of workers facing a major stressor in other location or settings. Our results seem to indicate that perceived organizational support goes beyond workload or access to mental help, and is different from social support. A thorough understanding of the distinction between these concepts and their specific impacts is a key to the success of future organizational initiatives. Finally, our study reinforces the notion of shared responsibility between individual, organizational and societal contributions regarding organizational and psychological health when facing a major stressor such as the COVID-19 pandemic [75, 76].

## Supplementary Information

**Additional file 1: Table S1.** Socio-demographic, occupational data, COVID-19 specific characteristics of participants who responded at both times-point surveys *(3 & 12-month surveys responders, n = 394).***Table S2.** Adjusted coefficient, 95% confidence interval and *p*-values from multivariable logistic regression model including self-compassion variable for burnout status among healthcare workers 12 months after the onset of COVID-19 pandemic *(12-month survey responders, n = 336; 74 missings)*. **Table S3.** Adjusted coefficient, 95% confidence interval and *p*-values from multivariable linear regression model including self-compassion variable for posttraumatic stress symptoms among healthcare workers 12 months after the onset of COVID-19 pandemic *(12-month survey responders, n = 343; 67 missings).***Table S4.** Adjusted coefficient, 95% confidence interval and *p*-values from multivariable linear regression model including self-compassion variable for anxiety symptoms among healthcare workers 12 months after the onset of COVID-19 pandemic *(12-month survey responders, n = 341; 69 missings).***Table S5.** Adjusted coefficient, 95% confidence interval and *p*-values from multivariable linear regression model including self-compassion variable for depression symptoms among healthcare workers 12 months after the onset of COVID-19 pandemic *(12-month survey responders, n = 341; 69 missings)*.

## Data Availability

In accordance with the ethical consent provided by participants, the data underlying this article cannot be shared publicly to preserve their privacy but are available from the corresponding author on reasonable request.
