# Supplementary material for: Evolution of burnout and psychological distress in healthcare workers during the COVID-19 pandemic: a 1-year observational study
Source: BMC Psychiatry. 2022 Dec 20;22:809. doi: 10.1186/s12888-022-04457-2 (PMC9763813; doi:10.1186/s12888-022-04457-2)
Supplement: Supplementary file 1 — Additional file 1: Table S1. Socio-demographic, occupational data, COVID-19 specific characteristics of participants who responded at both times-point surveys (3 & 12-month surveys responders, n = 394).Table S2. Adjusted coefficient, 95% confidence interval and p-values from multivariable logistic regression model including self-compassion variable for burnout status among healthcare workers 12 months after the onset of COVID-19 pandemic (12-month survey responders, n = 336; 74 missings). Table S3. Adjusted coefficient, 95% confidence interval and p-values from multivariable linear regression model including self-compassion variable for posttraumatic stress symptoms among healthcare workers 12 months after the onset of COVID-19 pandemic (12-month survey responders, n = 343; 67 missings).Table S4. Adjusted coefficient, 95% confidence interval and p-values from multivariable linear regression model including self-compassion variable for anxiety symptoms among healthcare workers 12 months after the onset of COVID-19 pandemic (12-month survey responders, n = 341; 69 missings).Table S5. Adjusted coefficient, 95% confidence interval and p-values from multivariable linear regression model including self-compassion variable for depression symptoms among healthcare workers 12 months after the onset of COVID-19 pandemic (12-month survey responders, n = 341; 69 missings). [file 12888_2022_4457_MOESM1_ESM.docx]

**Table S1.** Socio-demographic, occupational data, COVID-19 speciﬁc characteristics of participants who responded at both times-point surveys *(3 & 12-month surveys responders, n = 394)*

| **Variables** | **Mean ± *SD* or *n* (%)** | **All *n* = 394** |
| --- | --- | --- |
| **Age** (years) | 40 ± 9 | 393 |
| **Sex** (female) | 352 (90.7%) | 388 |
| **Ethnicity** |  | 394 |
| Caucasian | 375 (95.2%) |  |
| Hispanic | 1 (0.3%) |  |
| Black | 4 (1.0%) |  |
| Asian | 7 (1.8%) |  |
| Native American | 1 (0.3%) |  |
| Two of the above | 6 (1.5%) |  |
| **Marital status** |  | 392 |
| Never married | 66 (16.8%) |  |
| Married/Re-married | 117 (29.8%) |  |
| Separated/Divorced | 24 (6.1%) |  |
| Common-law union | 176 (44.9%) |  |
| Widowed | 1 (0.3%) |  |
| Other | 8 (2.0%) |  |
| **Parental status** (yes) | 252 (64.1%) | 393 |
| **Antecedent of psychiatric disorder** (yes) | 117 (29.7%) | 394 |
| **Work type** |  | 393 |
| Administrator | 17 (4.3%) |  |
| Administrative agent | 15 (3.8%) |  |
| Beneficiary attendant | 8 (2.0%) |  |
| Laboratory technician/technologist | 8 (2.0%) |  |
| Nurse | 87 (22.1%) |  |
| Other health professional (ergotherapist, respiratory therapist, psychologist, social worker, etc.) | 121 (30.8%) |  |
| Paramedics | 9 (2.3%) |  |
| Physician | 98 (24.9%) |  |
| Resident physician | 7 (1.8%) |  |
| Other | 23 (5.9%) |  |
| **Workplace** |  | 391 |
| Community clinic | 45 (11.5%) |  |
| Nursing home | 21 (5.4%) |  |
| University hospital | 121 (30.9%) |  |
| Non-University hospital | 71 (18.2%) |  |
| Medical clinic | 33 (8.4%) |  |
| Other | 100 (25.6%) |  |
| **Intensive care or emergency work** | 57 (14.6%) | 391 |
| **Workload** (hours/week) |  | 361 |
| ≤ 34 | 76 (21.1%) |  |
| 35-44 | 199 (55.1%) |  |
| 45-54 | 58 (16.1%) |  |
| 55-64 | 14 (3.9%) |  |
| ≥ 65 | 14 (3.9%) |  |
| **Current work status** |  | 379 |
| Still employed in the Quebec health system | 361 (95.3%) |  |
| Employee of another employer | 5 (1.3%) |  |
| Self-employed | 2 (0.5%) |  |
| Unemployed | 0 (0.0%) |  |
| Student | 2 (0.5%) |  |
| Retired | 2 (0.5%) |  |
| Other | 7 (1.8%) |  |
| **Access to mental help** (yes) | 364 (93.1%) | 391 |
| **Type of mental help professional** |  | 364 |
| Psychologist | 89 (24.5%) |  |
| Psychotherapist | 16 (4.4%) |  |
| Social worker | 6 (1.6%) |  |
| Family doctor | 47 (12.9%) |  |
| Employee assistance program | 175 (48.1%) |  |
| Other | 31 (8.5%) |  |
| **Access to PPE** |  | 389 |
| Never or rarely | 10 (2.6%) |  |
| Sometimes | 13 (3.3%) |  |
| Often | 70 (18.0%) |  |
| Always | 296 (76.1%) |  |
| **Perception of security using PPE** |  | 385 |
| Totally safe | 67 (17.4%) |  |
| Pretty safe | 284 (73.8%) |  |
| Rather or totally in danger | 34 (8.8%) |  |
| **Participation in simulation-based education** (yes) | 105 (27.0%) | 389 |
| **Last simulation session** |  | 105 |
| < 1 week | 2 (1.9%) |  |
| < 1 month | 4 (3.8%) |  |
| 1-2 months ago | 9 (8.6%) |  |
| < 6 months | 29 (27.6%) |  |
| < 1 year | 61 (58.1%) |  |
| **COVID status** |  | 392 |
| Negative | 334 (85.2%) |  |
| Recovered | 26 (6.6%) |  |
| Never been tested | 32 (8.2%) |  |
| **Direct COVID patient care** (yes) | 164 (41.9%) | 391 |
| **Reassignment** (yes) | 138 (35.1%) | 393 |

*Note*: Data are presented as Mean ± *SD* or *n* (%).

Abbreviations: PPE, personal protective equipment.

**Table S2.** Adjusted coefficient, 95% confidence interval and p-values from multivariable logistic regression model including self-compassion variable for burnout status among healthcare workers 12 months after the onset of COVID-19 pandemic *(12-month survey responders, n = 336; 74 missings)*

| **Variables** | | **OR** | **95% CI** | | ***p*** |
| --- | --- | --- | --- | --- | --- |
| **Independent** | **Resilience** | 0.66 | 0.49 | 0.90 | 0.009 |
|  | **Social support** | 0.71 | 0.54 | 0.94 | 0.02 |
|  | **Workload** |  |  |  | 0.13 |
|  | [35-44] h vs ≤ 34h | 1.65 | 0.89 | 3.06 | 0.11 |
|  | [45-54] h vs ≤ 34h | 1.82 | 0.81 | 4.10 | 0.15 |
|  | [55-64] h vs ≤ 34h | 3.67 | 0.86 | 15.67 | 0.08 |
|  | ≥ 65h vs ≤ 34h | 4.83 | 1.11 | 21.00 | 0.04 |
|  | **Perceived organizational support** | 0.66 | 0.51 | 0.85 | 0.001 |
|  | **Access to simulation technique** (yes vs no) | 0.89 | 0.52 | 1.52 | 0.66 |
|  | **Access to mental health help** (yes vs no) | 0.74 | 0.25 | 2.19 | 0.59 |
|  | **Access to PPE** | |  |  | 0.39 |
|  | Sometimes vs Never or rarely | 6.98 | 0.56 | 87.88 | 0.13 |
|  | Often vs Never or rarely | 4.67 | 0.68 | 31.84 | 0.12 |
|  | Always vs Never or rarely | 3.77 | 0.57 | 24.81 | 0.17 |
|  | **PPE perception of security** | | |  | 0.29 |
|  | Pretty safe vs Totally safe | 1.60 | 0.85 | 3.03 | 0.15 |
|  | Rather in danger or totally at risk vs Totally safe | 1.98 | 0.66 | 5.94 | 0.23 |
| **Adjustment** | **Psychiatric antecedent** (yes vs no) | 1.82 | 1.05 | 3.15 | 0.03 |
|  | **Self-compassion** | 0.99 | 0.74 | 1.32 | 0.94 |

*Note:* ORs are presented for an increase of one standard deviation (*SD*) for continuous variables (resilience; *SD* = 5.73, social support; *SD* = 5.69, perceived organizational support; *SD* = 11.53, and self-compassion; *SD* = 3.61).

Abbreviations: CI, Confidence intervals; OR, Odds ratio; PPE, Personal protective equipment.

**Table S3.** Adjusted coefficient, 95% confidence interval and *p*-values from multivariable linear regression model including self-compassion variable for posttraumatic stress symptoms among healthcare workers 12 months after the onset of COVID-19 pandemic *(12-month survey responders, n = 343; 67 missings)*

| **Variables** | | **Coefficient** | **95% CI** | | ***p*** |
| --- | --- | --- | --- | --- | --- |
| **Independent** | **Resilience** | -0.19 | -0.37 | -0.004 | 0.046 |
|  | **Social support** | -0.23 | -0.39 | -0.07 | 0.005 |
|  | **Workload** |  |  |  | 0.50 |
|  | [35-44] h vs ≤ 34h | -0.25 | -0.61 | 0.12 | 0.19 |
|  | [45-54] h vs ≤ 34h | 0.01 | -0.47 | 0.49 | 0.96 |
|  | [55-64] h vs ≤ 34h | 0.11 | -0.69 | 0.92 | 0.78 |
|  | ≥ 65h vs ≤ 34h | 0.06 | -0.74 | 0.85 | 0.89 |
|  | **Perceived organizational support** | -0.04 | -0.19 | 0.12 | 0.64 |
|  | **Access to simulation based education** (yes vs no) | -0.02 | -0.34 | 0.30 | 0.89 |
|  | **Access to mental health help** (yes vs no) | 0.33 | -0.31 | 0.97 | 0.31 |
|  | **Access to PPE** |  |  |  | 0.12 |
|  | Sometimes vs Never or rarely | 0.31 | -1.06 | 1.69 | 0.65 |
|  | Often vs Never or rarely | -0.51 | -1.56 | 0.54 | 0.34 |
|  | Always vs Never or rarely | -0.69 | -1.72 | 0.33 | 0.19 |
|  | **PPE perception of security** | |  |  | 0.0006 |
|  | Pretty safe vs Totally safe | 0.28 | -0.10 | 0.66 | 0.15 |
|  | Rather or totally in danger vs Totally safe | 1.23 | 0.60 | 1.87 | 0.0001 |
| **Adjustment** | **Psychiatric antecedent** (yes vs no) | 0.45 | 0.13 | 0.77 | 0.006 |
|  | **Self-compassion** | -0.21 | -0.38 | -0.04 | 0.02 |

*Note*: Regression coefficients are presented for an increase of one standard deviation (*SD*) for continuous variables (resilience; *SD* = 6.09, social support; *SD* = 5.93, perceived organizational support; *SD* = 11.65, and self-compassion; *SD* = 3.60).

Abbreviations: CI, Confidence intervals; PPE, Personal protective equipment.

**Table S4.** Adjusted coefficient, 95% confidence interval and p-values from multivariable linear regression model including self-compassion variable for anxiety symptoms among healthcare workers 12 months after the onset of COVID-19 pandemic *(12-month survey responders, n = 341; 69 missings)*

| **Variables** | | **Coefficient** | **95% CI** | | ***p*** |
| --- | --- | --- | --- | --- | --- |
| **Independent** | **Resilience** | -1.17 | -1.63 | -0.70 | <.0001 |
|  | **Social support** | -0.62 | -1.02 | -0.21 | 0.003 |
|  | **Workload** |  |  |  | 0.25 |
|  | [35-44] h vs ≤ 34h | -0.02 | -0.95 | 0.92 | 0.97 |
|  | [45-54] h vs ≤ 34h | 0.64 | -0.58 | 1.86 | 0.30 |
|  | [55-64] h vs ≤ 34h | 0.66 | -1.38 | 2.70 | 0.53 |
|  | ≥ 65h vs ≤ 34h | 1.98 | -0.11 | 4.06 | 0.06 |
|  | **Perceived organizational support** | -0.06 | -0.46 | 0.33 | 0.76 |
|  | **Access to simulation technique** (yes vs no) | 0.37 | -0.48 | 1.21 | 0.39 |
|  | **Access to mental health help** (yes vs no) | 0.41 | -1.22 | 2.03 | 0.62 |
|  | **Access to PPE** |  |  |  | 0.46 |
|  | Sometimes vs Never or rarely | 1.95 | -1.52 | 5.42 | 0.27 |
|  | Often vs Never or rarely | 0.59 | -2.06 | 3.24 | 0.66 |
|  | Always vs Never or rarely | 0.16 | -2.43 | 2.75 | 0.90 |
|  | **PPE perception of security** |  |  |  | 0.047 |
|  | Pretty safe vs Totally safe | 0.42 | -0.54 | 1.39 | 0.39 |
|  | Rather in danger or totally at risk vs Totally safe | 1.98 | 0.39 | 3.58 | 0.01 |
| **Adjustment** | **Intensive care of emergency work** (yes vs no) | 1.19 | 0.14 | 2.24 | 0.03 |
|  | **Psychiatric antecedent** (yes vs no) | 1.13 | 0.32 | 1.94 | 0.007 |
|  | **Self-compassion** | -0.38 | -0.82 | 0.05 | 0.08 |

*Note*: Regression coefficients are presented for an increase of one standard deviation (*SD*) for continuous variables (resilience; *SD* = 6.09, social support; *SD* = 5.93, perceived organizational support; *SD* = 11.65, and self-compassion; *SD* = 3.60).

Abbreviations: CI, Confidence intervals; PPE, Personal protective equipment.

**Table S5.** Adjusted coefficient, 95% confidence interval and *p*-values from multivariable linear regression model including self-compassion variable for depression symptoms among healthcare workers 12 months after the onset of COVID-19 pandemic *(12-month survey responders, n = 341; 69 missings)*

| **Variables** | | **Coefficient** | **95% CI** | | ***p*** |
| --- | --- | --- | --- | --- | --- |
| **Independent** | **Resilience** | -0.93 | -1.30 | -0.56 | <.0001 |
|  | **Social support** | -0.85 | -1.18 | -0.53 | <.0001 |
|  | **Workload** |  |  |  | 0.06 |
|  | [35-44] h vs ≤ 34h | -0.26 | -1.02 | 0.50 | 0.50 |
|  | [45-54] h vs ≤ 34h | 0.62 | -0.37 | 1.60 | 0.22 |
|  | [55-64] h vs ≤ 34h | 0.09 | -1.56 | 1.75 | 0.91 |
|  | ≥ 65h vs ≤ 34h | 1.69 | 0.06 | 3.31 | 0.04 |
|  | **Perceived organizational support** | -0.26 | -0.58 | 0.06 | 0.11 |
|  | **Access to simulation based education** (yes vs no) | -0.21 | -0.87 | 0.45 | 0.53 |
|  | **Access to mental health help** (yes vs no) | 0.65 | -0.66 | 1.96 | 0.33 |
|  | **Access to PPE** |  |  |  | 0.07 |
|  | Sometimes vs Never or rarely | 1.76 | -1.05 | 4.57 | 0.22 |
|  | Often vs Never or rarely | -0.58 | -2.72 | 1.56 | 0.60 |
|  | Always vs Never or rarely | -0.85 | -2.95 | 1.24 | 0.42 |
|  | **PPE perception of security** |  |  |  | 0.29 |
|  | Pretty safe vs Totally safe | 0.44 | -0.34 | 1.21 | 0.27 |
|  | Rather or totally in danger vs Totally safe | 1.01 | -0.28 | 2.29 | 0.13 |
| **Adjustment** | **Self-compassion** | -0.36 | -0.71 | -0.01 | 0.04 |

*Note*: Regression coefficients are presented for an increase of one standard deviation (*SD*) for continuous variables (resilience; *SD* = 6.09, social support; *SD* = 5.93, perceived organizational support; *SD* = 11.65, and self-compassion; *SD* = 3.60).

Abbreviations: CI, Confidence intervals; PPE, Personal protective equipment
